# Blocking Phosphatidylcholine Utilization in *Pseudomonas aeruginosa*, via Mutagenesis of Fatty Acid, Glycerol and Choline Degradation Pathways, Confirms the Importance of This Nutrient Source *In Vivo*

**DOI:** 10.1371/journal.pone.0103778

**Published:** 2014-07-28

**Authors:** Zhenxin Sun, Yun Kang, Michael H. Norris, Ryan M. Troyer, Mike S. Son, Herbert P. Schweizer, Steven W. Dow, Tung T. Hoang

**Affiliations:** 1 Department of Microbiology, University of Hawaii at Manoa, Honolulu, Hawaii, United States of America; 2 Department of Molecular Biosciences and Bioengineering, University of Hawaii at Manoa, Honolulu, Hawaii, United States of America; 3 Department of Biological Sciences, Plymouth State University, Plymouth, New Hampshire, United States of America; 4 Department of Microbiology, Immunology, and Pathology, Colorado State University, Fort Collins, Colorado, United States of America; University of Cambridge, United Kingdom

## Abstract

*Pseudomonas aeruginosa* can grow to very high-cell-density (HCD) during infection of the cystic fibrosis (CF) lung. Phosphatidylcholine (PC), the major component of lung surfactant, has been hypothesized to support HCD growth of *P. aeruginosa in vivo.* The phosphorylcholine headgroup, a glycerol molecule, and two long-chain fatty acids (FAs) are released by enzymatic cleavage of PC by bacterial phospholipase C and lipases. Three different bacterial pathways, the choline, glycerol, and fatty acid degradation pathways, are then involved in the degradation of these PC components. Here, we identified five potential FA degradation (Fad) related *fadBA*-operons (*fadBA1-5*, each encoding 3-hydroxyacyl-CoA dehydrogenase and acyl-CoA thiolase). Through mutagenesis and growth analyses, we showed that three (*fadBA145*) of the five *fadBA*-operons are dominant in medium-chain and long-chain Fad. The triple *fadBA145* mutant also showed reduced ability to degrade PC *in vitro*. We have previously shown that by partially blocking Fad, via mutagenesis of *fadBA5* and *fadD*s, we could significantly reduce the ability of *P. aeruginosa* to replicate on FA and PC *in vitro*, as well as in the mouse lung. However, no studies have assessed the ability of mutants, defective in choline and/or glycerol degradation in conjunction with Fad, to grow on PC or *in vivo*. Hence, we constructed additional mutants (Δ*fadBA145*Δ*glpD*, Δ*fadBA145*Δ*betAB*, and Δ*fadBA145*Δ*betAB*Δ*glpD*) significantly defective in the ability to degrade FA, choline, and glycerol and, therefore, PC. The analysis of these mutants in the BALB/c mouse lung infection model showed significant inability to utilize PC *in vitro*, resulted in decreased replication fitness and competitiveness *in vivo* compared to the complement strain, although there was little to no variation in typical virulence factor production (e.g., hemolysin, lipase, and protease levels). This further supports the hypothesis that lung surfactant PC serves as an important nutrient for *P. aeruginosa* during CF lung infection.

## Introduction

*Pseudomonas aeruginosa* is widespread in nature, inhabiting soil, water, plants and animals. In hospitals, it can be found in sinks, respirators, humidifiers and occasionally on the hands of medical personnel [1], [2]. The ubiquitous nature of this bacterium has allowed it to adapt to a broad range of hosts in which it can cause diseases. The role of *P. aeruginosa* as an opportunistic human pathogen is of particular concern, especially because it is a frequent cause of nosocomial infections such as pneumonia, urinary tract infections, and bacteremia [1], [3], [4]. *P. aeruginosa* infection in the respiratory tract of cystic fibrosis (CF) patients causes a rapid deterioration in lung function and thus patient survival [5], [6]. The pathogenicity of *P. aeruginosa* infection in CF patients has been extensively studied in terms of biofilm production [7]–[9] and quorum sensing (QS) controlled virulence [10]–[12]. However, little effort has been placed towards the contribution of *P. aeruginosa* nutrient acquisition aids high-cell-density (HCD) replication during lung infection.

Previous studies have shown that *P. aeruginosa* can undergo HCD replication in the lung of CF patients reaching >10^9^ CFU/ml [13]–[15]. HCD replication is highly energy demanding, requiring efficient nutrient acquisition and metabolism. However, evidence showed that the nutrients in the lung environment are lipids and amino acids derived from proteins or polypeptides [16]–[18], to allow *P. aeruginosa* HCD growth and maintenance *in vivo*. An *in vitro* study revealed that *P. aeruginosa* has directional twitching motility toward phosphatidylethanolamine (PE) and phosphatidylcholine (PC) [19]. Mammalian lungs are naturally coated by indispensible lung surfactant, which is composed of approximately 10% protein and 90% lipids, with about 80% of the lung surfactant lipids being phosphatidylcholine (PC) [20]–[22]. Thus, PC, the most abundant lipid in lung surfactant may provide significant nutrient for HCD bacterial growth *in vivo*. In accordance with this hypothesis, our initial studies suggest that PC is a major nutrient source of *P. aeruginosa* during lung infection and supports HCD replication [15], [17], [18].

**Figure 1:**
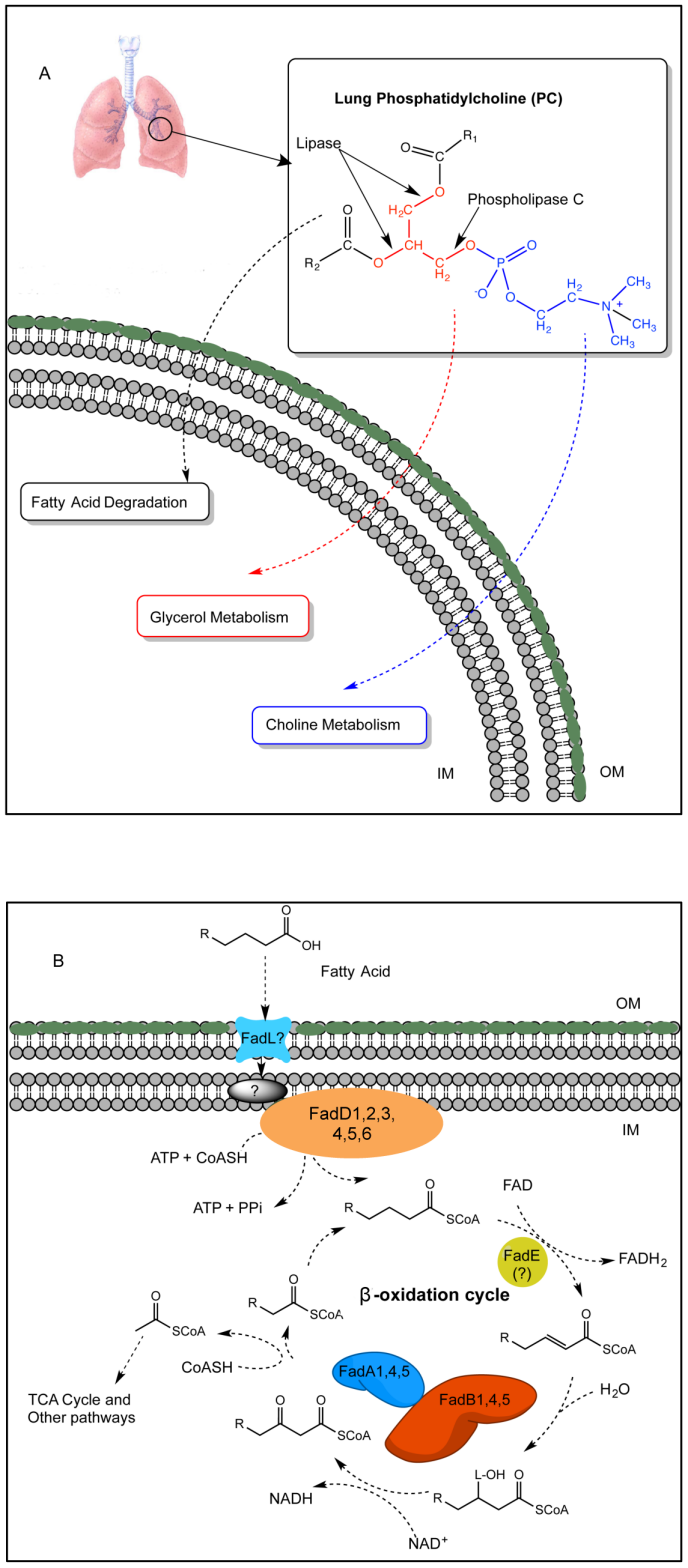
Phosphatidylcholine (PC) degradation pathways in *Pseudomonas aeruginosa*. (A) PC is the main component of lung surfactant and can be cleaved by phospholipase C and lipases, producing free fatty acids, glycerol, and phosphorylcholine. Three different pathways then further metabolize each component: the *bet* pathway for choline head group metabolism, the *glp* pathway for glycerol metabolism, and the *β*-oxidation pathway for the degradation of the FAs. (B) The proposed *P. aeruginosa* FA *β*-oxidation pathway. Abbreviations: FadA, 3-ketoacyl-CoA thiolase; FadB, *cis-*Δ^3^-*trans*-Δ^2^-enoyl-CoA isomerase, enoyl-CoA hydratase, 3-hydroxyacyl-CoA epimerase, and 3-hydroxyacyl-CoA dehydrogenase; FadD, fatty acyl-CoA synthetase; FadE, acyl-CoA dehydrogenase; FadL, outer membrane long-chain fatty acid translocase; OM, out membrane; IN, inner membrane.

Our previous *in vivo* CF sputa study showed that *P. aeruginosa* produced phospholipase C (heat-labile hemolysin) and lipases that could cleave exogenous PC into three components: a phosphorylcholine headgroup, glycerol and two long-chain fatty acids (LCFAs) [15] (Fig. 1A). These three components can be further metabolized by the choline (bet), glycerol (glp), and fatty acid degradation (Fad) pathways (Fig. 1B), respectively. Choline and glycerol metabolism by *P. aeruginosa* are well characterized [23]–[27]. However, LCFA degradation by *P. aeruginosa* and the genes involved in this process remain to be fully elucidated. Our previous *in vivo* CF sputa study also detected the expression of *P. aeruginosa* genes involved in each pathway for metabolizing all three PC components [15]. The *betAB-*operon was induced and *glpD* and *glpK* genes were constitutively expressed *in vivo* [15]. Among genes for Fad (Fig. 1B), the expression of *fadBA1* was detected when *P. aeruginosa* was grown on PC *in vitro,* and *fadBA5* and *fadA4* were induced and constitutively expressed in CF sputa [15], suggesting the involvement of *fadBA145* in LCFA degradation. We have also shown the reduced ability of the *fadD* mutants to utilize FAs as nutrients led to their decreased fitness during mouse lung infection [17], [18]. However, further evidence is needed to support the hypothesis that all three components of lung surfactant PC (phosphorylcholine, glycerol and FA) serve as nutrient sources for *P. aeruginosa* during *in vivo* lung infection.

In this study, up to five potential *fadBA*-operons (encoding 3-hydroxyacyl-CoA dehydrogenase and acyl-CoA thiolase) were identified, and three of them *fadBA1* (PA1737, PA1736), *fadBA4* (PA4786, PA4785) *and fadBA5* (PA3014, PA3013) were shown to be significantly involved in medium- and long-chain Fad. Coupling of the *fadBA145* mutations with mutations in choline and/or glycerol degradation were investigated to determine the importance of these pathways to degrade PC *in vitro* and *in vivo*. Competition studies were initiated to analyze the competitive fitness of these mutants relative to strains with intact pathways.

## Results and Discussion

### *P. aeruginosa* has five *fadBA*-operons potentially involved in fatty acid degradation

The well-established aerobic fatty acid degradation (Fad) pathway in *E. coli* was used as a model to characterize *P. aeruginosa* Fad. *E. coli* possesses only a single copy of each *fad* gene for aerobic Fad [28], [29], and the cyclic degradation of fatty acids by two carbons per cycle is primarily catalyzed by an acyl-CoA dehydrogenase coded by *fadE*, and the products of the *fadBA*-operon, 3-hydroxyacyl-CoA dehydrogenase and acyl-CoA thiolase, respectively. Up to five potential *fadBA*-operons were identified in *P. aeruginosa* by BLAST analysis of the *E.coli fadBA* sequence against the *P. aeruginosa* genome, including *fadBA1* (PA1737, PA1736), *fadBA2* (PA3590, PA3589), *fadBA3* (PA2554, PA2553), *fadBA4* (PA4786, PA4785), and *fadBA5* (PA3014, PA3013) (Fig. S1). Of these five FadBAs, FadBA5 showed the greatest homology to the *E.coli* FadBA with FadB5 having 72% similar (54% identical) and FadA5 having 76% similar (61% identical) to the *E.coli* FadBA, respectively [30], [31].

Considering the larger size of the *P. aeruginosa* genome (6.29 Mb) [32] and its wide range of environmental niches and metabolic capabilities, it is not surprising that *P. aeruginosa* has up to five *fadBA*-operon homologues. Therefore, it is important next to narrow down which of these five operons are important in Fad, using a mutagenesis approach.

### *P. aeruginosa fadBA145*-operons are important for degrading PC and medium- and long-chain fatty acids

Our previous work showed that the Δ*fadBA5* mutant has a reduced ability to utilize LCFA as a sole carbon source, but this Δ*fadBA5* mutant can still grow on LCFAs as a carbon source, indicating the existence of other potential *fadBA-*operons in *P. aeruginosa* for LCFA degradation [31]. Further supporting this idea, the *fadBA1-*operon was shown to be induced by medium-chain fatty acids (MCFAs) and to a lesser extent by LCFAs [33]. The *fadBA5-*operon plays the most significant role in LCFA degradation, because neither the single Δ*fadBA1* mutant, nor the single Δ*fadBA4* mutant, showed much defects in their ability to utilize MCFA and LCFA as sole carbon sources (Fig. S2). It is possible that the FadBA1 and/or other FadBA(s) might have overlapping functions with FadBA5 in the metabolism of different chain length FAs, but these activities are masked by the more dominant FadBA5. Evidence for the involvement of other FadBAs is lacking, and needs to be addressed.

**Figure 2:**
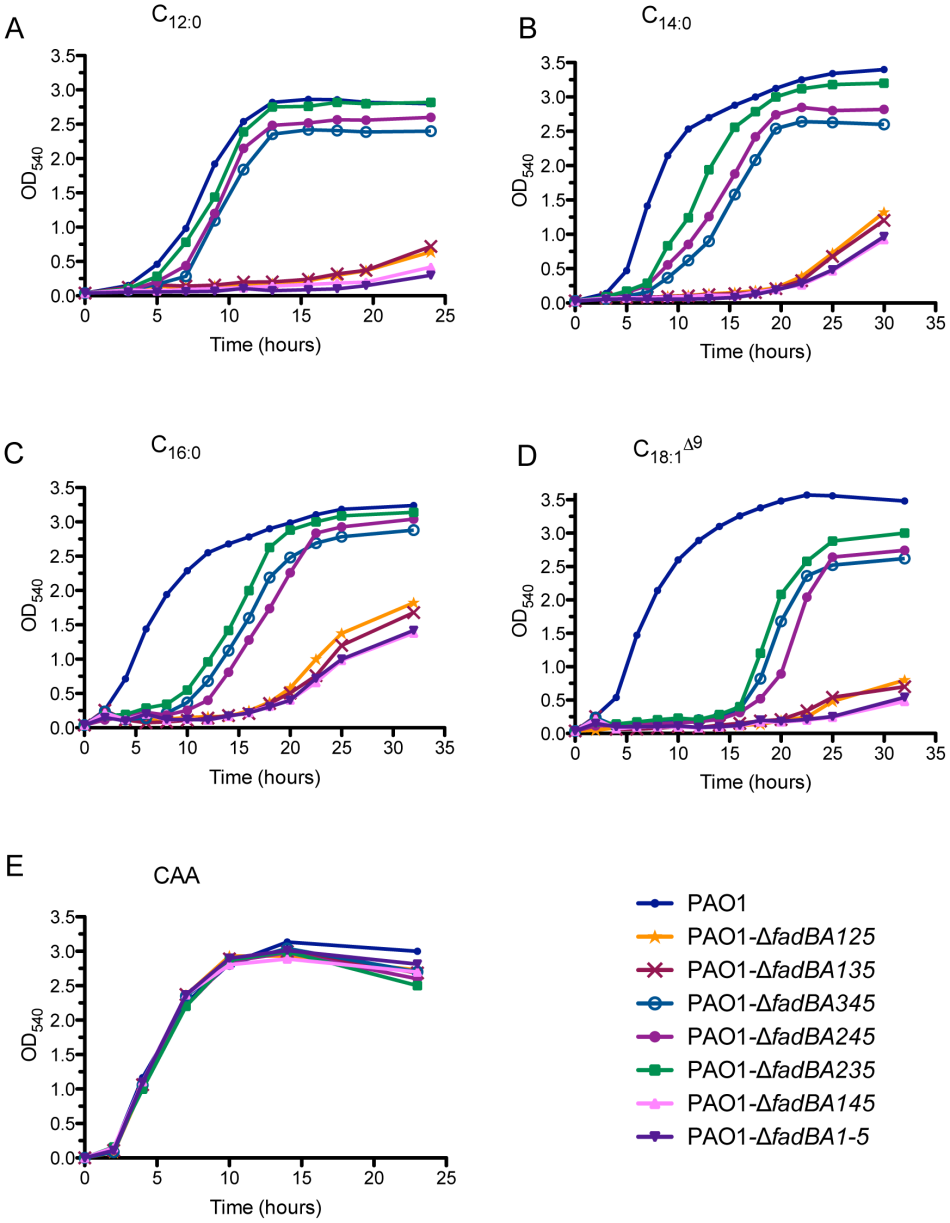
Growth analysis of different *fadBA* mutant combinations on medium (C_12∶0_) and long chain-length fatty acid (C_14∶0_, C_16∶0_ and C_18∶1_^Δ9^). Along with the wildtype PAO1 strain, mutants were grown in 1×M9 minimal medium supplemented with 0.4% different test FAs (A to D) or 1% control casamino acids (CAA, E) as sole carbon sources. Although *fadBA* mutants showed various defects when grown with FAs of different chain-lengths, no growth defects were observed for any of the mutants when grown with CAA as a control.

Because it is too overwhelming to test all possible double, triple, and quadruple *fadBA*-mutant combinations and the complicated dominance of *fadBA5*, we demonstrated the involvement of each *fadBA*-operon by testing different triple mutant combinations. In this study, we generated several triple mutants (Δ*fadBA125*, Δ*fadBA135,* Δ*fadBA145,* Δ*fadBA235,* Δ*fadBA245,* Δ*fadBA345*) and a quintuple mutant Δ*fadBA1-5* (Table 1) for growth analysis on MCFA and LCFAs as sole carbon sources, to further characterize the function of these *fadBA-*operons. The growth defects were previously defined by the slower growth rate and lower overall final cell densities compared to wildtype strain PAO1, which suggest a reduced ability to metabolize these FAs, presumably due to the accumulation of growth inhibiting intermediates [17], [31]. Significant growth defects were observed for any combinations of triple mutants in which both the *fadBA5*- and *fadBA1-*operons were deleted (i.e., Δ*fadBA125,* Δ*fadBA135* and Δ*fadBA145* mutants), revealing the importance of *fadBA1* and *fadBA5* contributing to growth on MCFA and LCFAs (Fig. 2A–D). Although the level of defects is different for each type of FA used, the trend is consistent for these mutants in all FAs (Fig. 2A–D). Only the Δ*fadBA145* triple mutant showed the same growth defect as the Δ*fadBA1-5* quintuple mutant on all fatty acids tested, suggesting the importance of all three *fadBA1*, *fadBA4*, and *fadBA5* operons and the minor role of *fadBA2* and *fadBA3*-operons in metabolizing MCFA and LCFAs. The importance of *fadBA4* was further confirmed by the fact that the Δ*fadBA245* and Δ*fadBA345* triple mutants showed an additional growth defect on all FAs compared to the Δ*fadBA235* mutant (Fig. 2A–D). However, the *fadBA4-*operon displays less of an involvement in metabolizing all FAs tested compared to both *fadBA1* and *fadBA5-*operons (i.e., comparing Δ*fadBA245* to Δ*fadBA125* or Δ*fadBA345* to Δ*fadBA135*). Knowing that only *fadBA1*, *fadBA4*, and *fadBA5* are important for degrading MCFA and LCFA of PC, we continued with further experiments from this point forward in our study by using the Δ*fadBA145* mutant background, rather than the Δ*fadBA1-5* quintuple mutant.

**Table 1:** Bacterial strains used in this study^a^.

Strain	Lab ID^b^	Genotype/Description	Reference
*E.coli*			
EP-Max10B	E1231	F*^−^* λ*^−^ mcrA* Δ(*mrr-hsdRMS-mcrBC*) φ80d*lacZ* Δ*M15* Δ*lacX74 deoR recA1* *endA1 araD139* Δ(*ara, leu*)7697 *galU galKrpsL nupG*	BioRad
SM10	E006	*thi thr leu tonA lacY supE recA*::RP4-2Tc::Mu Km^r^	[48]
*P. aeruginosa*			
PAO1	P007	Prototroph	[49]
PAO1- *mucA^−^*	P447	Cb^r^, PAO1 with pUC18 inserted in *mucA* gene	This study
Δ*fadBA125*	P122	Gm^r^, PAO1-Δ*fadBA1*::*FRT,* Δ*fadBA2*::*FRT,* Δ*fadBA5*::Gm	This study
Δ*fadBA135*	P124	Gm^r^, PAO1-Δ*fadBA1*::*FRT,* Δ*fadBA3*::*FRT,* Δ*fadBA5*::Gm	This study
Δ*fadBA145*	P319	Gm^r^, PAO1-Δ*fadBA1*::*FRT,* Δ*fadBA4*::*FRT,* Δ*fadBA5*::Gm	This study
Δ*fadBA235*	P317	Gm^r^, PAO1-Δ*fadBA2*::*FRT,* Δ*fadBA3*::*FRT,* Δ*fadBA5*::Gm	This study
Δ*fadBA245*	P130	Gm^r^, PAO1-Δ*fadBA2*::*FRT,* Δ*fadBA3*::*FRT,* Δ*fadBA5*::Gm	This study
Δ*fadBA345*	P126	Gm^r^, PAO1-Δ*fadBA3*::*FRT,* Δ*fadBA4*::*FRT,* Δ*fadBA5*::Gm	This study
Δ*fadBA1-5*	P102	Gm^r^, PAO1-Δ*fadBA1*::*FRT,* Δ*fadBA2*::*FRT,* Δ*fadBA3*::*FRT,*Δ*fadBA4*::*FRT,* Δ*fadBA5*::Gm	This study
Δ*fadBA145* Δ*glpD*	P539	Gm^r^, PAO1-Δ*fadBA1*::*FRT,* Δ*fadBA4*::*FRT,* Δ*fadBA5*::*FRT,*Δ*glpD*::Gm*-FRT1*	This study
Δ*fadBA145* Δ*betAB*	P555	Gm^r^, PAO1-Δ*fadBA1*::*FRT,* Δ*fadBA4*::*FRT,* Δ*fadBA5*::*FRT,*Δ*betAB*::Gm*-FRT3*	This study
Δ*fadBA145* Δ*betAB* Δ*glpD*	P561	Gm^r^, PAO1-Δ*fadBA1*::*FRT,* Δ*fadBA4*::*FRT,* Δ*fadBA5*::*FRT,*Δ*betAB*::*FRT3,* Δ*glpD*::Gm*-FRT1*	This study
Δ*fadBA145/*complement	P965	Gm^r^, Tet^r^ *;* Δ*fadBA145* complemented with miniCTX2*-fadBA5*	This study
Δ*fadBA145* Δ*glpD/*complement	P1015	Gm^r^, Tet^r^ *;* Δ*fadBA145* Δ*glpD* complemented with miniCTX2*-fadBA5/glpD*	This study
Δ*fadBA145* Δ*betAB/*complement	P1017	Gm^r^, Tet^r^ *;* Δ*fadBA145* Δ*betAB* complemented with miniCTX2*-fadBA5/betAB*	This study
Δ*fadBA145* Δ*betAB*Δ*glpD/*complement	P1019	Gm^r^, Tet^r^ *;* Δ*fadBA145* Δ*betAB* Δ*glpD* complemented withminiCTX2*-fadBA5/betAB/glpD*	This study
Δ*fadBA145-mucA^−^*	P576	Gm^r^, Cb^r^; Δ*fadBA145* with pUC18 inserted in *mucA* gene	This study
Δ*fadBA145* Δ*glpD-mucA^−^*	P570	Gm^r^, Cb^r^; Δ*fadBA145* Δ*glpD* with pUC18 inserted in *mucA* gene	This study
Δ*fadBA145* Δ*betAB-mucA^−^*	P572	Gm^r^, Cb^r^; Δ*fadBA145* Δ*betAB* with pUC18 inserted in *mucA* gene	This study
Δ*fadBA145* Δ*betAB*Δ*glpD-mucA^−^*	P574	Gm^r^, Cb^r^; Δ*fadBA145* Δ*betAB* Δ*glpD* with pUC18 inserted in *mucA* gene	This study
Δ*fadBA145-* *mucA^−/^*complement	P584	Gm^r^, Cb^r^, Tet^r^; Δ*fadBA145-mucA^−^* complemented with miniCTX2*-fadBA5*	This study
Δ*fadBA145* Δ*glpD-* *mucA^−/^*complement	P578	Gm^r^, Cb^r^, Tet^r^; Δ*fadBA145* Δ*glpD-mucA^−^* complemented withminiCTX2*-fadBA5/glpD*	This study
Δ*fadBA145* Δ*betAB-* *mucA^−/^*complement	P580	Gm^r^, Cb^r^, Tet^r^; Δ*fadBA145* Δ*betAB-mucA^−^* complemented withminiCTX2*-fadBA5/betAB*	This study
Δ*fadBA145* Δ*betAB* Δ*glpD-* *mucA^−/^*complement	P582	Gm^r^, Cb^r^, Tet^r^; Δ*fadBA145* Δ*betAB* Δ*glpD-mucA^−^* complemented withminiCTX2*-fadBA5/betAB/glpD*	This study

a For strains constructed in this study, please see text for further details.

b Please use Lab ID for requesting strains.

**Figure 3:**
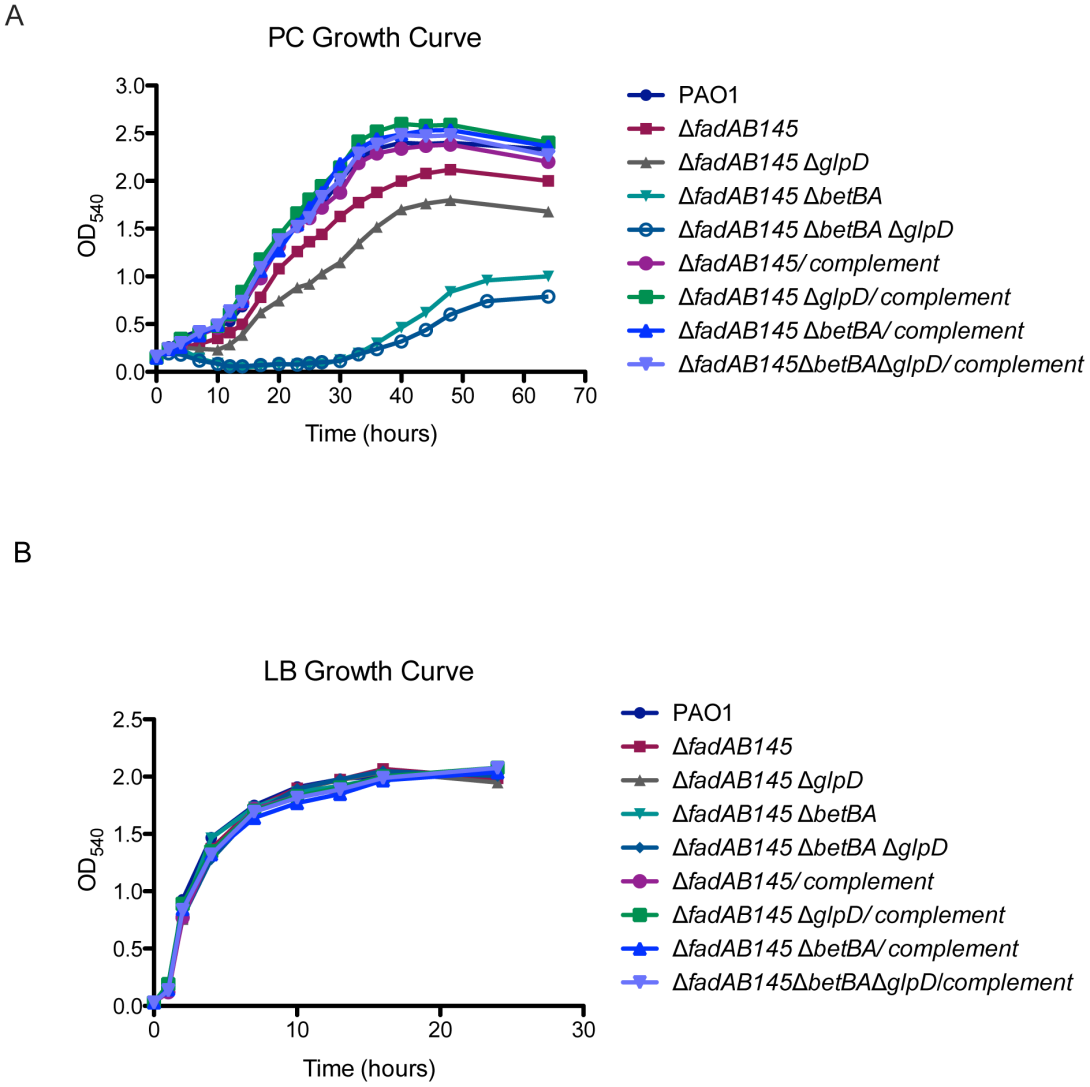
Growth analysis on phosphatidylcholine. (A) Some mutants exhibited growth defects on PC as a sole carbon source. The growth defects were fully recovered in complemented strains, as they had identical growth rates compared to the wildtype PAO1 strain. (B) No growth defects in control LB medium were observed.

**Figure 4:**
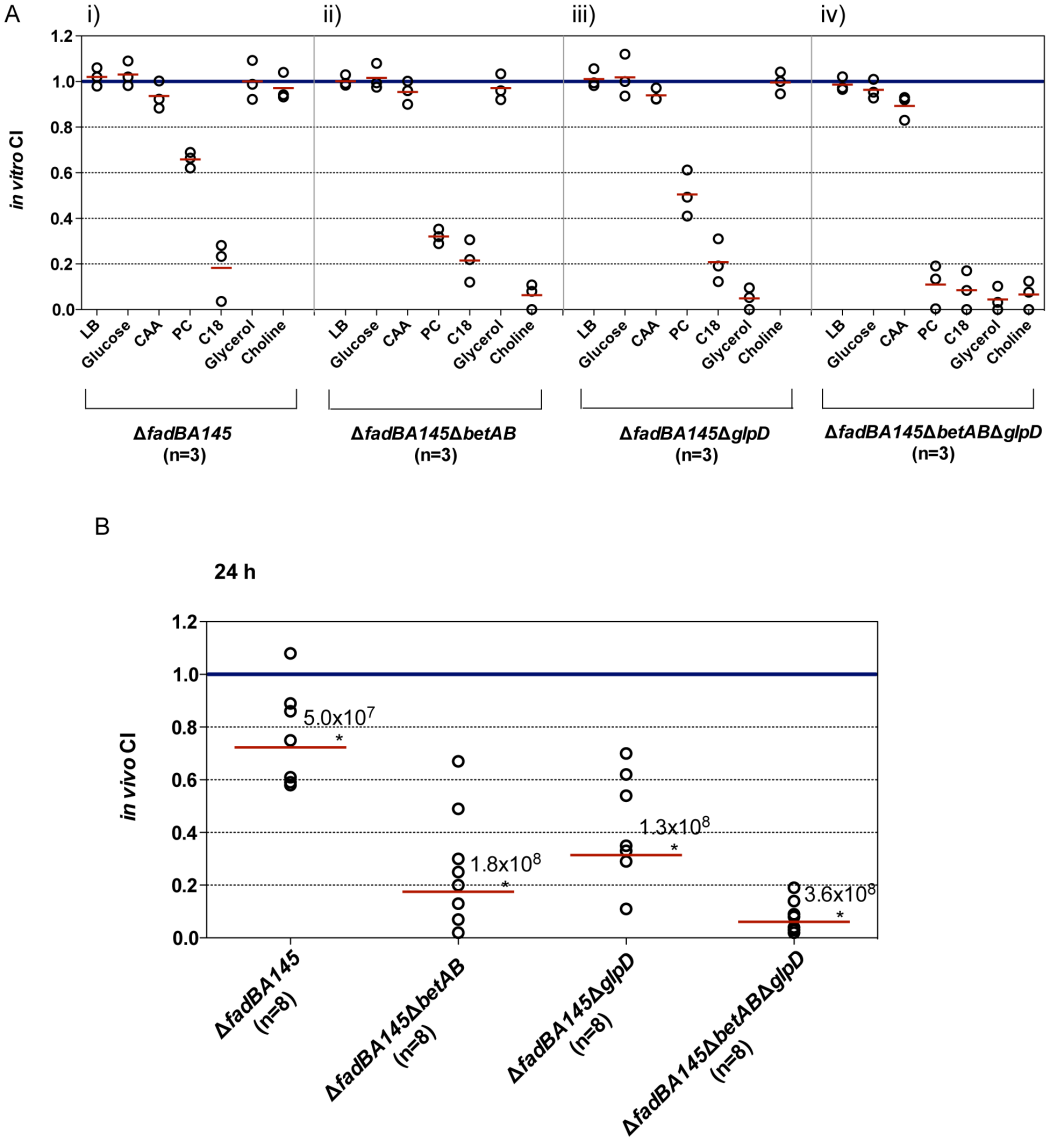
Competition studies of pathway mutants. (A) *In vitro* competition studies of the various mutants and their complemented strains in different growth media (n equals the number of independent *in vitro* competition experiments performed with each carbon source). (B) *In vivo* lung competition of the various mutants and their complemented strains after 24 h, where n equals the number of mice in each group that were inoculated with a total of 6×10^6^ CFU/mouse. The solid red line indicates the geometric mean of the competitive indices (CI) in each competition group. CI<1 indicates the mutant was less competitive than its complemented strain in various growth media (A) or within the lungs (B). Numbers above the red line represent the average total recovered CFU/mouse for each competition group.

We next tested the Δ*fadBA145* mutant for its ability to grow on PC. The Δ*fadBA145* triple mutant displayed a reduced growth rate and lower final cell density as compared to wildtype PAO1 when grown with PC as a sole carbon source (Fig. 3A). There was no growth defect observed for this mutant when grown in control LB medium (Fig. 3B). The Δ*fadBA145* mutant strain is competitively less fit than the complement in the *in vitro* competition study when grown in PC or FA (C_18∶1_^Δ9^) (Fig. 4A panel i). No competitive defect was observed when the Δ*fadBA145* mutant was grown on LB, glucose, casamino acids (CAA), glycerol or choline controls (Fig. 4A panel i). We concluded that the reduced growth of the Δ*fadBA145* mutant strain on PC observed in figure 3A is due to a decreased ability to degrade LCFAs of PC and not glycerol or choline. All the evidence we have here strongly suggests the involvement of the three *fadBA1,4,5-*operons in Fad and PC degradation. We complemented Δ*fadBA145* mutant strain by integrating miniCTX2-*fadBA5* (i.e., the dominant *fadBA* operon as explained above) as a single copy into the Δ*fadBA145* mutant background (Table 1). The complemented strain was fully restored to wildtype growth on PC (Fig. 3A) and FAs (not shown). Hence, all complementation experiments in this study for any Δ*fadBA145* mutant background were performed with only the dominant *fadBA5*-operon.

### Mutants blocked in FA, glycerol, and choline degradation displayed dramatically reduced ability to utilize PC *in vitro*

The enzymatic activity of phospholipase C on PC releases the phosphorylcholine headgroup and the diacylglycerol (DAG) molecule (Fig. 1A). The phosphorylcholine headgroup is first transported across the cell membrane and dephosphorylated by a phosphatase [23], [34], [35] to yield choline, which has previously been shown to be sufficient for *P. aeruginosa* to grow on as a sole carbon, nitrogen, and energy source [36]. *P. aeruginosa* BetAB (encoding choline dehydrogenase and a glycine betaine aldehyde dehydrogenase) catalyzes the conversion of choline to glycine betaine [23]. Glycine betaine is successively demethylated to form dimethylglycine (DMG), sarcosine (monomethylglcine), and finally glycine [24], [37]. The DAG molecule is cleaved by the *P. aeruginosa* lipase, liberating a glycerol molecule and two LCFAs. Glycerol metabolism has been well characterized in *P. aeruginosa*. The operon primarily consists of *glpD* (a *sn*-glycerol-3-phosphate dehydrogenase [38]), *glpF* (a membrane-associated glycerol diffusion facilitator [27], [39]), *glpK* (a glycerol kinase [27], [39]), *glpM* (a membrane protein affecting alginate synthesis [26]), and *glpR* (a regulator of the *glp* operon [25]).

Since our previous data showed that *betAB* and *glpD* were expressed *in vivo* [15], they may potentially be involved in PC degradation during lung infection. However, before we could address the *in vivo* aspect of PC degradation, further experiments are needed to characterize *P. aeruginosa* PC degradation *in vitro*. We engineered double pathway mutants Δ*fadBA145*Δ*betAB* (blocked in FA and choline degradation) and Δ*fadBA145*Δ*glpD* (blocked in FA and glycerol degradation) and a triple pathway mutant Δ*fadBA145*Δ*betAB*Δ*glpD* (blocked in FA, choline, and glycerol degradation) (Table 1), to further determine whether these mutants are deficient in growth on PC. As expected, all three mutants experienced various growth defects with decreased cell density and delayed log-phase when grown on PC (Fig. 3A). The triple pathway mutant Δ*fadBA145*Δ*betAB*Δ*glpD* exhibited the most significant reduced ability to utilize PC, reaching to only about one-third of the wildtype final cell density. We complemented these mutants (i.e., Δ*fadBA145*Δ*betAB*, Δ*fadBA145*Δ*glpD*, and Δ*fadBA145*Δ*betAB*Δ*glpD*) by integrating the respective miniCTX2*-fadBA5/betAB*, miniCTX2*-fadBA5/glpD* or miniCTX2*-fadBA5/betAB/glpD* as a single copy on the *P. aeruginosa* chromosome and the complemented strains fully recovered these mutants’ ability to grow on PC as compared to wildtype PAO1 (Fig. 3A). No mutants or complement showed any growth defects on control LB medium (Fig. 3B).

We performed an *in vitro* competition study between pathway mutants (Δ*fadBA145*Δ*betAB,* Δ*fadBA145*Δ*glpD,* Δ*fadBA145*Δ*betAB*Δ*glpD*) and their complements to exam whether the mutation reduced their ability to metabolize various carbon sources. As expected, all the pathway mutants were less competitive than their respective complements in media containing PC and other sole carbon sources involved in the respective pathways (Fig. 4A panels ii-iv). For example, the Δ*fadBA145*Δ*betAB* mutant was less competitive than its complement when grown using PC, FA, or choline, as sole carbon source (Fig. 4A panel ii), which is not the case in other control media (e.g., LB, glucose, CAA or glycerol). Likewise, the Δ*fadBA145*Δ*glpD* mutant was less competitive than its complement only if PC, FA, or glycerol was used as sole carbon source (Fig. 4A panel iii). The triple pathway mutant was almost completely outcompeted by its complemented strain with the competitive indices (CI) dramatically reduced to ∼0.1 when growing in the media containing PC, choline, glycerol, or oleate FA (Fig. 4A panel iv). Overall, these *in vitro* data showed that we have three mutants (Δ*fadBA145*Δ*betAB,* Δ*fadBA145*Δ*glpD,* Δ*fadBA145*Δ*betAB*Δ*glpD*) and their complement that could be used to assess the utilization of PC *in vivo*, through competitive index experiments within the mouse lung.

### Blocking FA, glycerol, and choline degradation significantly reduces replication fitness of *P. aeruginosa in vivo*

Since PC is the major component of lung surfactant in mammals, including mice [21], a mouse lung infection model [40] was utilized for our *in vivo* competition study to evaluate the fitness of the PC degradation pathway mutants within the lung environment. The mucoid, exopolysaccharide alginate-overproducing phenotype is a distinguishing feature of *P. aeruginosa* isolated from CF patients [40], [41]. An alginate-overproducing strain carrying a *mucA* insertional mutation, which allows the mucoid strain to survive and replicate in the lung, has been successfully used in BALB/c mouse model to establish the connection between nutrient acquisition and *in vivo* lung fitness for *P. aeruginosa* [17]. Therefore, we constructed various *mucA^−^* alginate-overproducing strains, such as Δ*fadBA145-mucA^−^*, Δ*fadBA145*Δ*glpD-mucA^−^*, Δ*fadBA145*Δ*betAB-mucA^−^*, Δ*fadBA145*Δ*betAB*Δ*glpD-mucA^−^* and their complemented strains for this study. Prior to the animal study, the phenotypes of all *mucA* strains were confirmed by patching on minimal media plates with FA, choline, or glycerol as sole carbon sources along with all appropriate controls (all *mucA* wild-type strains and complemented strains). As expected, the *mucA* mutation did not affect the metabolism of any of these carbon sources (Fig. S3). As previously described [17], [40], BALB/c mice were inoculated via intratracheal intubation with equal ratios of each mutant and its complement pair (6×10^6^ CFU/animal). At 24 h post-infection, bacterial CFU recovered from the lungs were determined, followed by CI calculations. For all the strains, the average total CFU per mouse lung recovered at 24 hours post-infection was greater than the initial inoculum (Fig. 4B), indicating that all these *P. aeruginosa* strains maintained the ability to replicate within the mouse lung. The Δ*fadBA145* mutant still replicated significantly *in vivo* compared to its complement. Surprisingly, the Δ*fadBA145* CI is quite high compared to other FAD mutants (i.e., *fadD* mutants) we have previously published where CI is approximately 0.5 [17], [18]. The Δ*fadBA145*Δ*betAB*, Δ*fadBA145*Δ*glpD in vivo* CI is lower than the Δ*fadBA145* when compared to their respective complements (Fig. 4B), showing the importance of glycerol and choline degradation as potential nutrient sources *in vivo*. Most significantly, the mean CI value for the triple pathway mutant (i.e., Δ*fadBA145*Δ*betAB*Δ*glpD*) showed that the triple pathway mutant had a significantly reduced ability to survive and multiply in the lungs of mice compared to its complement.

We monitored all strains tested *in vivo* for different virulence expression, including proteases, rhamnolipid, hemolysins and lipases (Fig. S4). With similar level of these common secreted virulence factors observed between strains (Fig. S2), the low CI is most likely due to its inability to metabolize PC and the three components of PC (LCFAs, glycerol, and phosphorylcholine) as a nutrient source, rather than resulting from altered virulence expression. Overall, the altered ability for the pathway mutants to metabolize PC as nutrient *in vitro* was clearly mirrored by their competitive fitness within the lung.

In summary, *P. aeruginosa* possesses an impressive repertoire of virulence factors, and the expression of most of these only occurs during the HCD replication and their timely expression is regulated by QS [42], which occurs at HCD. *P. aeruginosa* requires large amount of readily available energy to reach and maintain HCD and produce the high-energy dependent virulence structure like biofilm. Thus, exploration of the nutrient sources supporting such an energy intensive processes is of importance, especially for chronic *P. aeruginosa* lung infections in CF patients. In addition, the identification of the genes and pathways for *P. aeruginosa* HCD replication in CF lungs provides fundamental knowledge for possibly developing new therapeutic strategies targeting bacterial nutrient metabolism in the lung, thereby preventing bacterial HCD. The expression of genes involved in *P. aeruginosa* PC degradation within the lungs of CF patients has been previously demonstrated [15]. Our study focused on providing further evidence to determine whether PC serves as a significant nutrient source during *P. aeruginosa* lung infection. In order to decipher the role of PC *in vivo,* we first characterized PC degradation pathways *in vitro*. Of the three components released by the enzymatic cleavage of PC by bacterial phospholipase C and lipases (phosphorylcholine, LCFAs, and glycerol), LCFAs are highly reduced and yield the most carbon and energy. In our study, five potential *fadBA-*operons were investigated and three of them (i.e., *fadBA1,4,5-*operons) proved to be significantly involved in Fad. The *in vitro* growth analysis of different pathway mutants (Δ*fadBA145,* Δ*fadBA145*Δ*betAB,* Δ*fadBA145*Δ*glpD,* Δ*fadBA145*Δ*betAB*Δ*glpD*) on PC provided direct evidence to support that *P. aeruginosa* utilizes the FA, glycerol and choline degradation pathways to degrade individual components of PC *in vitro.* Our *in vivo* competition study was performed utilizing a mouse lung infection model [40] to evaluate the fitness of the pathway mutants within the lung environment. The triple pathway mutant Δ*fadBA145*Δ*betAB*Δ*glpD* exhibited the greatest growth defect on relevant carbon sources *in vitro* and was outcompeted by its complement *in vivo*. Since no altered expression of virulence factors was observed for all the pathway mutants and their complement pairs compared to wildtype PAO1, it is highly likely that the decreased ability to utilize PC resulted in lower replication fitness in the lung environment. This study strongly supports the hypothesis that *P. aeruginosa* utilizes lung surfactant PC as one of the nutrient sources for chronic lung infection.

## Materials and Methods

### Ethic Statement

All animal experiments were performed in compliance with the NIH (National Institutes of Health) Guide for the Care and Use of Laboratory Animals and were approved by the University of Hawaii Institutional Animal Care and Use Committee (protocol no. 06-023-04).

### Bacterial strains and growth conditions

Bacterial strains and plasmids utilized in this study are listed in Table 1 and 2. *E. coli* EP-Max10B was used as cloning strains and cultured in Luria-Bertani (LB) medium (Difco). *Pseudomonas* Isolation Agar or Broth (PIA or PIB; Difco) or LB medium were used to culture *P. aeruginosa* strain PAO1 and derivatives. All fatty acids (FAs) stocks were made as previously described [17]. Strains for growth analyses were cultured in 1× M9 minimal medium +0.2% (w/v) Brij-58 (Sigma) +1% (w/v) casamino acids (CAA) or 0.4% (w/v) of the individual FA, C_12∶0_ to C_16∶0_, or C_18∶1_^Δ9^ (Sigma; Fig. 2) and 1× M9 minimal medium +0.2% (w/v) Brij-58 (Sigma) +0.4% (w/v) phosphatidylcholine (PC, Sigma; Fig. 3A), at 37°C with a shaking speed of 200 r.p.m. Since most of FAs hydrolyzed from *in vivo* PC are C_16∶0_ (50–60%), with ∼10–20% of each of C_14∶0_, C_16∶1_, C_18∶1_^Δ9^, and C_18∶2_ constituting the rest [43], the growth analysis was performed in 1×M9 minimal medium supplied with each of C_12∶0_ (medium-chain fatty acid), C_14∶0_, C_16∶0_, and C_18∶1_^Δ9^ (LCFAs) as a sole carbon source. Accordingly, the PC we used in these *in vitro* experiments contains mostly LCFAs, approximately 33% C_16∶0_, 13% C_18∶0_, 31% C_18∶1_^Δ9^, and 15% C_18∶2_. The *in vitro* competition studies (Fig. 4A) were performed under the growth condition mentioned above as previously described [17].

**Table 2:** Plasmids used in this study^a^.

Plasmids	Lab ID^b^	Relevant properties	Reference
pFlp2	E0067	Ap^r^, *sacB* ^+^; Flp-containing plasmid	[45]
pPS856	E0050	Ap^r^; Gm^r^; plasmid with Gm^r^-*FRT*-cassette	[45]
pUC18	E0135	Ap^r^; cloning vector	[50]
pUC18-‘*mucA*’	E1907	Ap^r^; *mucA* internal region cloned into pUC18	This study
pUC19	E0014	Ap^r^; cloning vector with P*_lac_*	[50]
pUC19-*glpD*	E1843	Ap^r^; pUC19 with *glpD* gene cloned in downstream of P*_lac_*	This study
pEX18T	E0055	Ap^r^, *ori*T^+^, *sacB* ^+^; gene replacement vector	[45]
pEX18TΔ*fadBA1*::Gm	E0202	Ap^r^, Gm^r^; pEX18T with *ΔfadBA1 operon with* Gm^r^-*FRT*-cassette insertion	This study
pEX18TΔ*fadBA2*::Gm	E0224	Ap^r^, Gm^r^; pEX18T with *ΔfadBA2 operon with* Gm^r^-*FRT*-cassette insertion	This study
pEX18TΔ*fadBA3*::Gm	E0225	Ap^r^, Gm^r^; pEX18T with *ΔfadBA3 operon with* Gm^r^-*FRT*-cassette insertion	This study
pEX18TΔ*fadBA4*::Gm	E0226	Ap^r^, Gm^r^; pEX18T with *ΔfadBA4 operon with* Gm^r^-*FRT*-cassette insertion	This study
pEX18TΔ*fadBA5*::Gm	E0461	Ap^r^, Gm^r^; pEX18T with *ΔfadBA5 operon with* Gm^r^-*FRT*-cassette insertion	This study
pEX18TΔ*glpD*::Gm	E1066	Ap^r^, Gm^r^; pEX18T with *ΔglpD operon with* Gm^r^-*FRT*-cassette insertion	This study
pEX18TΔ*betAB*::Gm	E1070	Ap^r^, Gm^r^; pEX18T with *ΔbetAB operon with* Gm^r^-*FRT*-cassette insertion	This study
miniCTX2	E0076	Tet^r^; site-specific integration vector	[46]
miniCTX2-*fadBA5*	E1765	Tet^r^; miniCTX2 with cloned *fadBA5*	This study
miniCTX2*-fadBA5/glpD*	E2035	Tet^r^; miniCTX2 with cloned *fadBA5/glpD*	This study
miniCTX2*-fadBA5/betAB*	E1953	Tet^r^; miniCTX2 with cloned *fadBA5/betAB*	This study
miniCTX2*-fadBA5/betAB/glpD*	E1992	Tet^r^; miniCTX2 with cloned *fadBA5/betAB/glpD*	This study

a For plasmids constructed in this study, please see text for further details.

b Please use Lab ID for requesting plasmids.

### General molecular methods

Oligonuceotides were synthesized through Integrated DNA Technology and are listed in Table 3. All molecular methods and their components utilized were employed as previously described [44].

**Table 3:** Primers used in this study.

Primer number and name	Sequence^a^
186; *fadBA1*-upstream	5′-CGAAAGCTTGCATGGTGCTATCTTCC-3′
187; *fadBA1*-downstream	5′-GCGGAATTCGCCCTACCCGTGGCG-3′
218; *fadBA2*-upstream	5′-CGGTGAAGCTTTCGCGCACC-3′
219; *fadBA2*-downstream	5′-GGGGAATTCGGTGTCTCATCGGCAGCGC-3′
220; *fadBA3*-upstream	5′-GCGAAGCTTATTCAGCAGGAGAAAACGACG-3′
221; *fadBA3*-downstream	5′-TGCGGAATTCGACGGATAGTCGCCGCTAC-3′
211; *fadBA4*-upstream	5′-CGTAAGCTTGCCGGGGAGTCAGGGGC-3′
212; *fadBA4*-downstream	5′-CCCGAATTCGCACGGCACCGCCCAAG-3′
272; *fadBA5-*HindIII	5′-AGTTCAAGCTTCCATAATAGC-3′
273; *fadBA5*-EcoRI	5′-CCCGGAATTCCCCCTTCGAGAACGCTTAG-3′
518; *glpK*-BamHI	5′-AGCTGAAGTGGATCCTCGACAA-3′
519; *glpKD-*SacI	5′-CTGGCGAGCTCAGGCCGCATGCACCCG-3′
522; *betA*-SacI	5′-CAACGAGCTCGGCGATATCTACGGCGG-3′
523; *betB*-HindIII	5′-GCCAAAGCTTCCAGGACAAGAACGGCT3′
888; Xho-*fadB5*	5′-CCTGCGCAGAGGGCCTCGAGGAGGGC-3′
889; *fadB5*-Bam	5′-GGGCACGAGGATCCCCGGCTTTCCCC-3′
895; Spe-*betB*	5′-CGGATTCAGACTAGTACCTGCTCG-3′
896; Hind-*glpD*	5′-GCCTGGTGAAGCTTCGGGCTGGTC-3′
927; SacI-P*_lac_*-*glpD*	5′-CGCTCGCCGGAGCTCGAACGACCGAGC-3′

a Restriction enzyme sites utilized in this study are underlined.

### Construction of mutants and complementation strains

All mutants were constructed as described previously [45]. Briefly, the *fadBA* (*fadBA1*, *fadBA2*, *fadBA3*, *fadBA4*, *fadBA5*) operons, *betAB* operon, and *glpD* gene were amplified by PCR using respective upstream and downstream primer pair listed in Table 3. The PCR products were purified from the gel, digested with appropriate restriction enzymes, and cloned into the gene replacement vector pEX18T, digested with the same restriction enzymes, to yield each of the pEX18T-target gene constructs. After deletions were made on plasmid in each of the *fadBA*-operons, the *glpD* gene, and the *betAB*-operon through restriction digestion (*fadBA1*: *Pst*I, *Bam*HI; *fadBA2*: *Stu*I, *Bam*HI; *fadBA3*: *Not*I, *Sma*I; *fadBA4*: *Eco*RV; *fadBA5*: *Sph*I, *Pst*I) and blunt-ended (except for *glpD*, which was blunt-ended using *SmaI*), the 1.1 kb *FRT*-Gm^R^-*FRT* cassette obtained from pPS856 digested with *Sma*I was inserted into each gene. These newly constructed pEX18T vectors were transformed into *E.coli* SM10 or ER2566-mob, and conjugated into PAO1 to engineer the unmarked mutations as previously described [45]. To obtain all triple mutants, we invested an enormous amount of work to first create all single and double mutants with the proper confirmed Flp/*FRT*-excision of the gentamycin antibiotic resistance cassette to recycle this resistance marker for subsequent mutagenesis (data not shown).

The single copy integration vector, miniCTX2, was used to engineer the complemented strains for each triple-pathway mutant as previously described [46]. Briefly, *fadBA5* and *betAB* were PCR amplified with primers 888/889 and 522/895, respectively. The miniCTX2-*fadBA5* was derived by inserting *fadBA5* fragment into miniCTX2, both digested with *Xho*I and *Bam*HI. The *betAB* gene was sub-cloned in using *Sac*I and *Spe*I, yielding miniCTX2*-fadBA5-betAB. glpD* was first cloned into pUC19 by digesting the PCR product with *Hind*III and *Sac*I, which was amplified using primers 896/519. P*_lac_*-*glpD* fragment was amplified using primers 519/927 from pUC19-*glpD* and cloned into miniCTX2-*fadBA5* and miniCTX2*-fadBA5-betAB* to yield miniCTX2*-fadBA5-glpD* and miniCTX2*-fadBA5-betAB-glpD,* respectively.

The newly engineered mutant strains Δ*fadBA145*, Δ*fadBA145*Δ*glpD*, Δ*fadBA145*Δ*betAB*, and Δ*fadBA145*Δ*betAB*Δ*glpD*, were complemented using the relevant gene(s) on the miniCTX2 single copy integration vector as preciously described [46]. The resulting strains Δ*fadBA145/*complement, Δ*fadBA145*Δ*glpD/*complement, Δ*fadBA145*Δ*betAB/*complement, Δ*fadBA145*Δ*betAB*Δ*glpD/*complement were used in the growth curve experiment (Table 1 and Fig. 3A).

### Growth characterization of mutants and complementation strains

Growth curve analyses have been described previously [17]. Briefly, all strains utilized were initially grown overnight in *Pseudomonas* Isolation Broth (PIB). The overnight cultures were centrifuged and the cell pellets were washed twice with 1×M9 minimal medium, and then a 1∶50 dilution was made into 25 ml of the respective media (described above) for different growth curves. To clarify any insoluble FA, individual cultures were diluted 4-fold in 4% Brij-58, pre-incubated at 42°C for 2 minutes, prior to taking OD_540_ measurement at each time point (Fig. 2 and 3). To obtain the growth curves in Figure S2, all of the strains were grown overnight at 37°C in LB broth. The overnight cultures were centrifuged and the cell pellets were washed twice with 1×M9 minimal medium, and then a 1∶400 dilution was made into respective media (described above) for different growth curves. 125 µl aliquots of the diluted cultures were transferred to a sterile, polystyrene 96-well assay plate (Falcon *Microtest flat bottom* plate, catalog no. *35–1172*; Becton-Dickinson Labware). Growth was recorded using an ELx808 Absorbance Microplate Reader (BioTek Instruments, Winooski, VT) under the following conditions: temperature37°C, and shaking at a low speed. The plate was read at 630 nm every 30 min for 40 h. All of the data was transferred and plotted using Prism.

### Virulence factors detection

Strains used for virulence factors detection were grown in LB medium. At each time point, aliquots of individual culture were used for OD_540_ measurement (Fig. 3B). The detection of proteases, hemolysins, lipases, and rhamnolipid was performed as described elsewhere [17]. All assays were conducted in triplicate, and the data were analyzed as previously described [17].

### Growth Phenotype Confirmation of Mucoid and Non-mucoid Strains

To confirm that mutations in *mucA* do not have additional effects on nutrient metabolism of the pathway mutant strains, all of the pathway mutants and complement strains were purified on LB plate or LB plate supplemented with 250 µl/ml carbenicillin (Cb250) for *mucA^−^* strains. After 24 h incubation at 37°C, single colony of each strain was patched on 1× M9 solid medium +1% (w/v) Brij-58 supplemented with 0.2% (w/v) C_18∶1_^Δ9^, 40 mM glycerol or 30 mM choline as sole carbon source. They were also patched on LB plate, which served as a control. The growth pattern was observed after 24–36 h incubation at 37°C (Fig. S3).

### *In vitro* and *in vivo* competition studies

*In vitro* and *in vivo* competition studies were performed as previously described [17]. Briefly, seven growth media with different carbon sources, including Luria-Bertani (LB) medium, casamino acids (CAA), glucose, PC, C_18∶1_^Δ9^, choline, and glycerol, were used in this study. The bacterial CFU were determined after inoculation into each of the medium for 24–48 h. The CI was calculated as the CFU ratio of mutant/wildtype recovered at each time point divided by the CFU ratio of mutant/wildtype in the input inoculum [47]. The smaller the CI value, the more the significant reduction in fitness of the mutant.

Various alginate-overproducing strains, Δ*fadBA145-mucA^−^*, Δ*fadBA145*Δ*glpD-mucA^−^*, Δ*fadBA145*Δ*betAB-mucA^−^*, Δ*fadBA145*Δ*betAB*Δ*glpD-mucA^−^* and the complement strains for each mutant utilized in this study are listed in Table 1. The use of the *mucA^−^* mutation is essential in this animal model as previously described [40].

## Supporting Information

Figure S1 **Five potential** ***fadBA*** **-operon homologues of** ***P. aeruginosa.*** (A) Genes of operons (GenBank accession numbers in parentheses) are shown in light purple with percent of identity and similarity to the *E. coli* FadBA. *fadBA1* is 3.363 kb; *fadBA2* is 2.760 kb; *fadBA3* is 2.346 kb; *fadBA4* is 2.887 kb; and *fadBA5* is 3.353 kb, (B) Alignment of *P. aeruginosa* FadAs and FadBs with *E.* coli FadA and FadB motifs. Amino acids with similar properties are assigned the same colors using CLC Sequence Viewer 6.
(TIF)

Figure S2 **Growth analysis of different single** ***fadBA*** **mutants on medium (C_12∶0_) and long chain-length fatty acid (C_14∶0_, C_16∶0_ and C_18∶1_^Δ9^).** Along with the wildtype PAO1 strain, mutants Δ*fadBA1*, Δ*fadBA2*, Δ*fadBA3*, Δ*fadBA4* and Δ*fadBA5* were grown in 1×M9 minimal medium supplemented with 0.05% different test FAs (A to D) and 1% Brij-58 or LB broth as a control (E). Only the Δ*fadBA5* mutant showed various defects when grown with FAs of different chain-lengths, no significant growth defects were observed for the rest of single *fadBA* mutants. All of the mutants grew to the same level as wildtype when grown in LB.
(TIF)

Figure S3 **Growth Phenotype Confirmation of Mucoid and Non-mucoid Strains.** Along with the wildtype PAO1 and PAO1-*mucA^−^* strains, all of the pathway mutants and their corresponding complement strains were patched on 1× M9 solid medium +1% (w/v) Brij-58 supplemented with 0.2% (w/v) C_18∶1_^Δ9^ (B), 40 mM glycerol (C), or 30 mM choline (D). (A) Growth on LB was performed as a control. Alginate over-producing strains show a light sheen surface indicated by white arrow in panel A. Similar growth defects were shown between mucoid and non-mucoid strains on different plates. A detailed plate layout is shown in panel E with strains identification of Table 1 in parentheses.
(TIF)

Figure S4 **Analyses of proteases, hemolysins, lipases, and rhamnolipid productions by** ***P. aeruginosa*** **various pathway mutant.** No mutants displayed significant (*P*≤0.05, based on student *t*-test) decrease in productions of proteases (A), rhamnolipid (B), hemolysins (C), and lipases (D).
(TIF)
