# Supplementary figures and images for: Blocking Phosphatidylcholine Utilization in Pseudomonas aeruginosa, via Mutagenesis of Fatty Acid, Glycerol and Choline Degradation Pathways, Confirms the Importance of This Nutrient Source In Vivo
Source: PLoS One. 2014 Jul 28;9(7):e103778. doi: 10.1371/journal.pone.0103778 (PMC4113454; doi:10.1371/journal.pone.0103778)

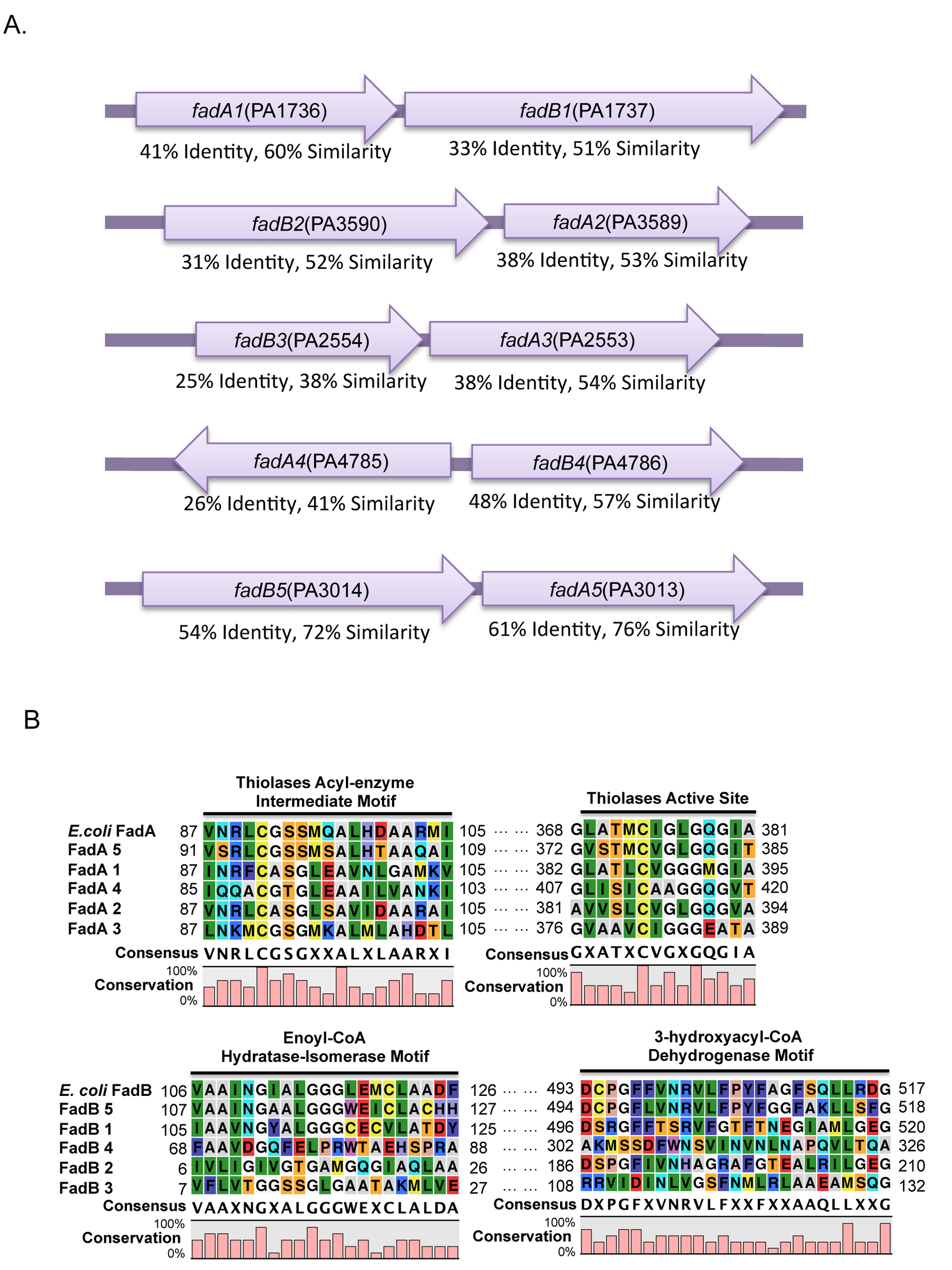

Supplement: Figure S1 — Five potential fadBA -operon homologues of P. aeruginosa. (A) Genes of operons (GenBank accession numbers in parentheses) are shown in light purple with percent of identity and similarity to the E. coli FadBA. fadBA1 is 3.363 kb; fadBA2 is 2.760 kb; fadBA3 is 2.346 kb; fadBA4 is 2.887 kb; and fadBA5 is 3.353 kb, (B) Alignment of P. aeruginosa FadAs and FadBs with E. coli FadA and FadB motifs. Amino acids with similar properties are assigned the same colors using CLC Sequence Viewer 6. (TIF) [file pone.0103778.s001.tif]

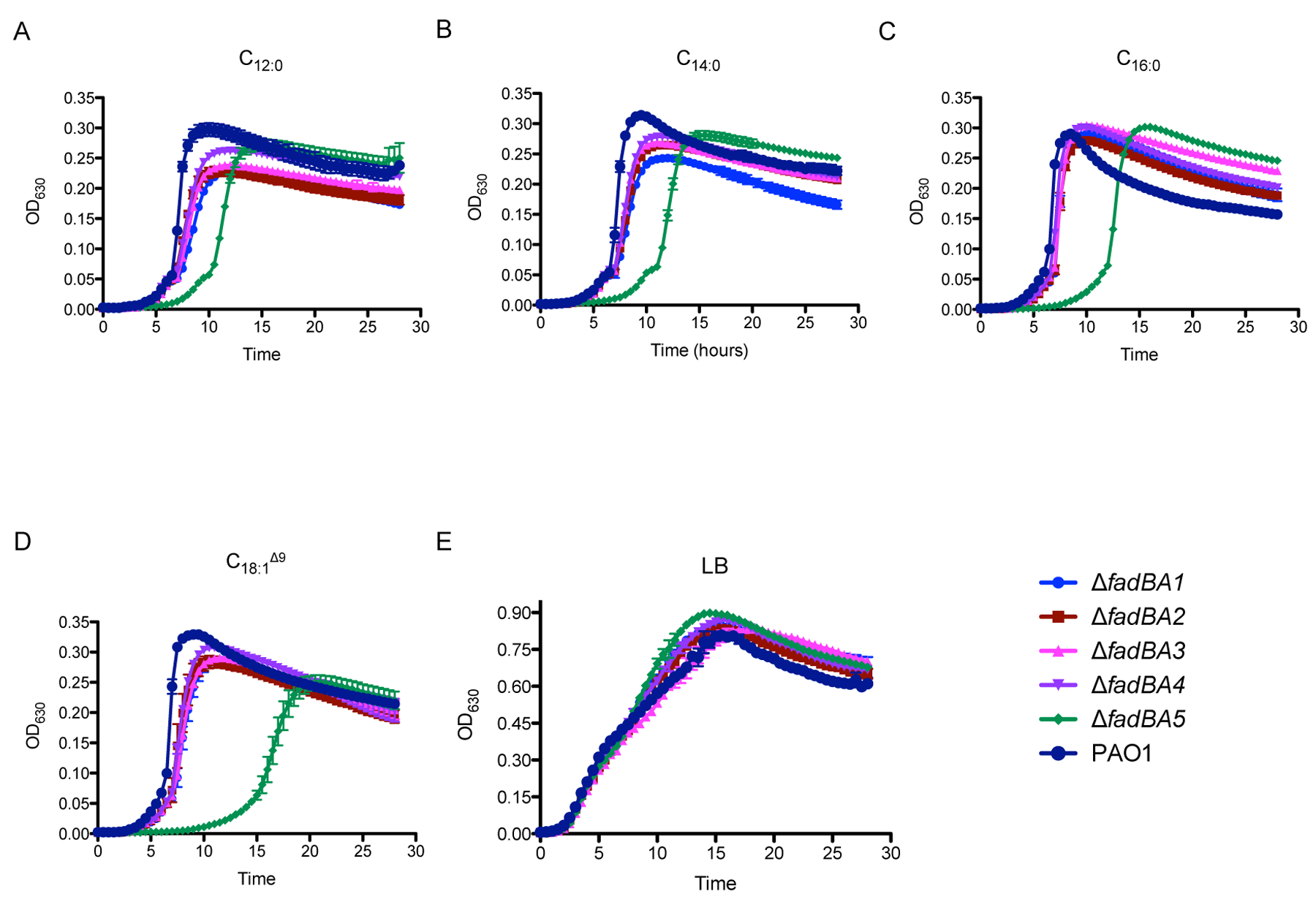

Supplement: Figure S2 — Growth analysis of different single fadBA mutants on medium (C12∶0) and long chain-length fatty acid (C14∶0, C16∶0 and C18∶1Δ9). Along with the wildtype PAO1 strain, mutants ΔfadBA1, ΔfadBA2, ΔfadBA3, ΔfadBA4 and ΔfadBA5 were grown in 1×M9 minimal medium supplemented with 0.05% different test FAs (A to D) and 1% Brij-58 or LB broth as a control (E). Only the ΔfadBA5 mutant showed various defects when grown with FAs of different chain-lengths, no significant growth defects were observed for the rest of single fadBA mutants. All of the mutants grew to the same level as wildtype when grown in LB. (TIF) [file pone.0103778.s002.tif]

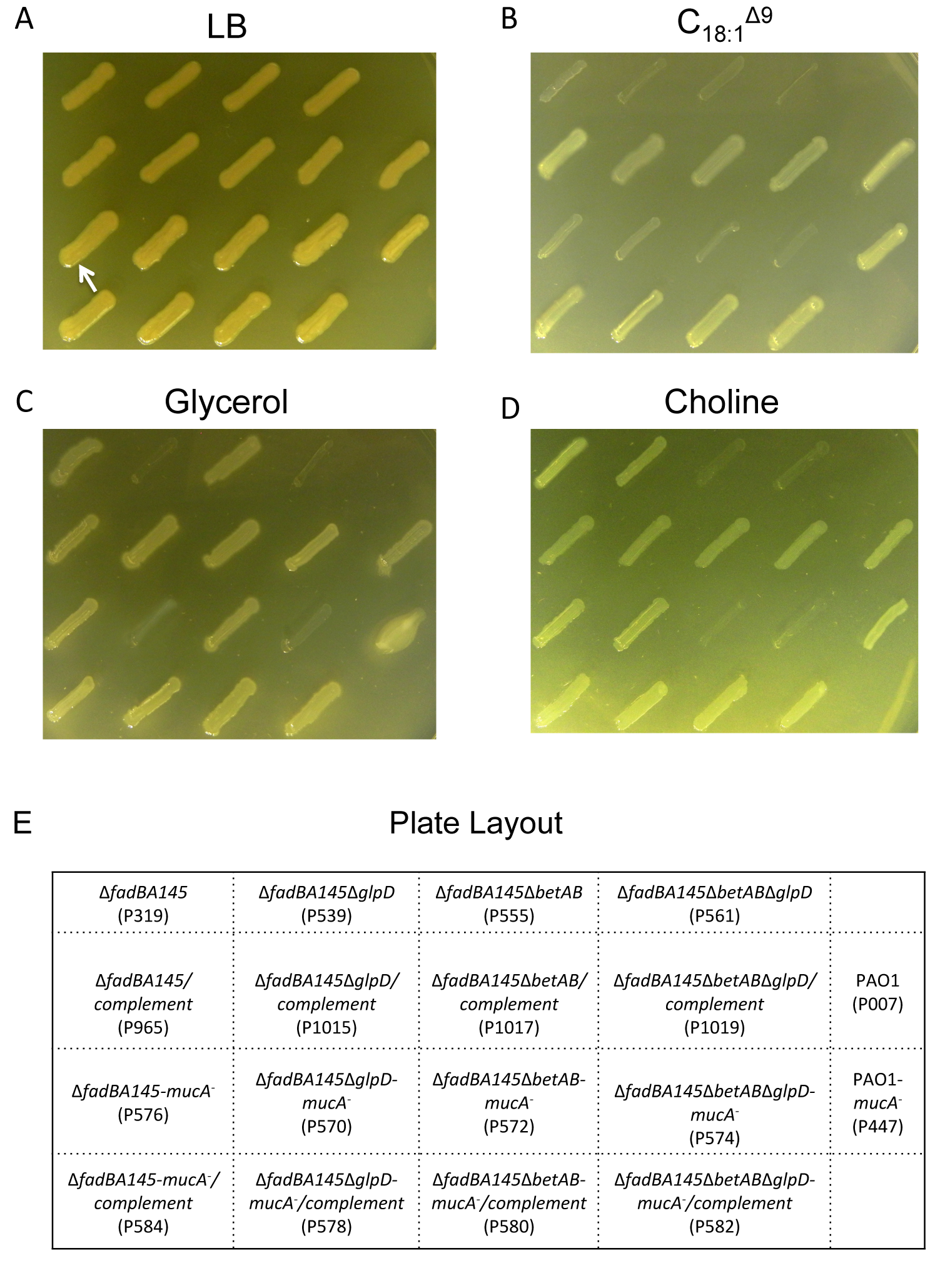

Supplement: Figure S3 — Growth Phenotype Confirmation of Mucoid and Non-mucoid Strains. Along with the wildtype PAO1 and PAO1-mucA− strains, all of the pathway mutants and their corresponding complement strains were patched on 1× M9 solid medium +1% (w/v) Brij-58 supplemented with 0.2% (w/v) C18∶1 Δ9 (B), 40 mM glycerol (C), or 30 mM choline (D). (A) Growth on LB was performed as a control. Alginate over-producing strains show a light sheen surface indicated by white arrow in panel A. Similar growth defects were shown between mucoid and non-mucoid strains on different plates. A detailed plate layout is shown in panel E with strains identification of Table 1 in parentheses. (TIF) [file pone.0103778.s003.tif]

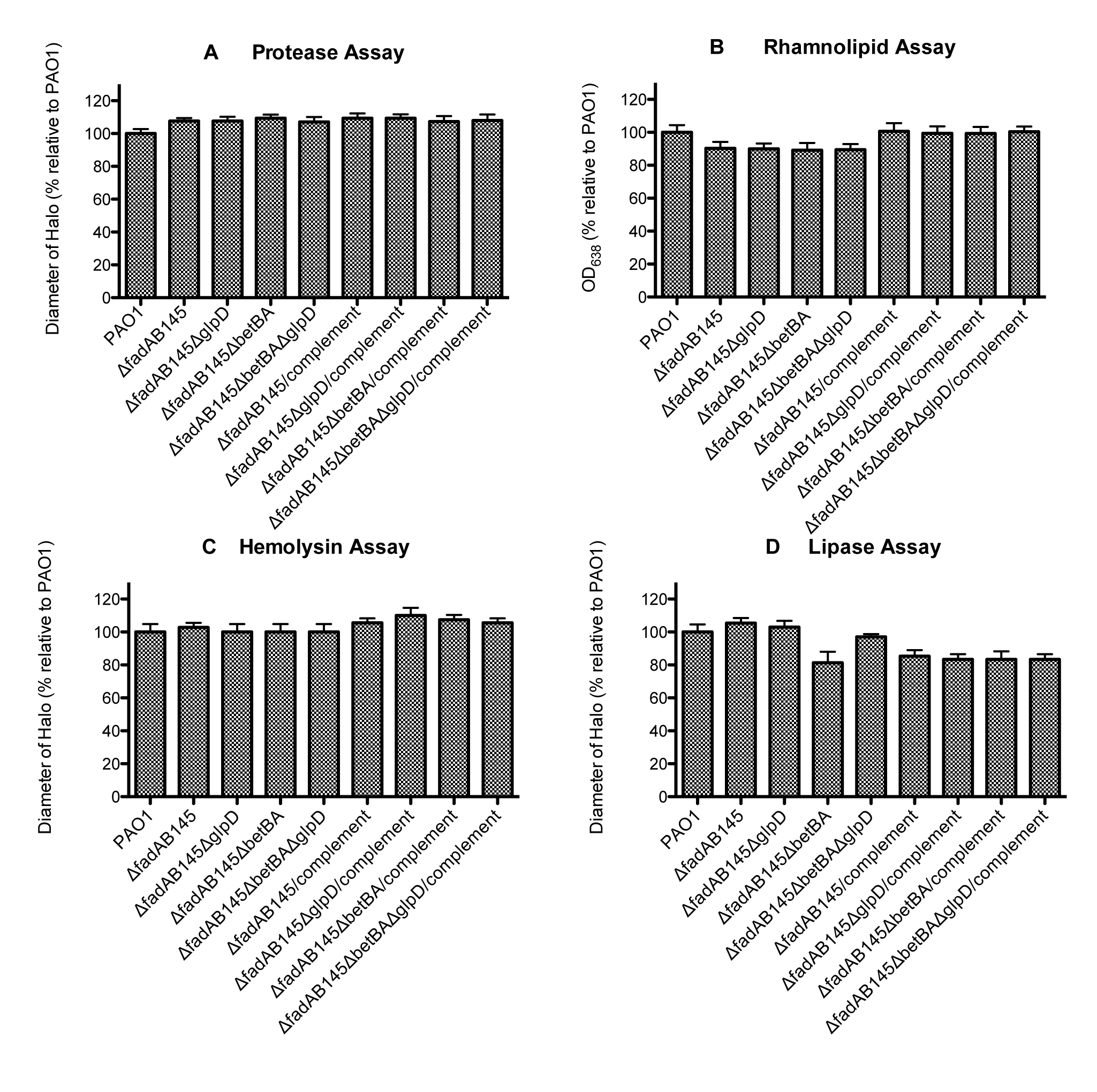

Supplement: Figure S4 — Analyses of proteases, hemolysins, lipases, and rhamnolipid productions by P. aeruginosa various pathway mutant. No mutants displayed significant (P≤0.05, based on student t-test) decrease in productions of proteases (A), rhamnolipid (B), hemolysins (C), and lipases (D). (TIF) [file pone.0103778.s004.tif]
